# Site identification and selection: current practices within the trial managers network and NIHR chief investigators

**DOI:** 10.1186/1745-6215-16-S2-P175

**Published:** 2015-11-16

**Authors:** Dawn Coleby, Diane Whitham, Lelia Duley

**Affiliations:** 1 Nottingham Clinical Trials Unit, University of Nottingham, Nottingham, UK

## Background

Selecting sites is an important part of a clinical trial. Selection of poor sites can lead to reduced recruitment and lower quality of data. Currently there are no tools available to assess either suitability of a potential site, or factors that could be addressed to improve suitability.

In order to develop a ‘site selection tool’ to improve site selection, we surveyed Trial Managers and Chief Investigators to ask about their current practice for choosing sites in their trial. The aim was to identify ‘key performance factors’.

## Method

A short online survey was distributed to all members of the UK Trial Managers Network and NIHR Chief Investigators. The questions asked about: use of any method for site selection; if and how potential sites are assessed for suitability; the definition of a successful site; and if and how site performance is assessed. Free text responses were assessed to identify themes, and grouped into ‘key performance factors’ which could be used to assess the suitability of a site for inclusion into a trial.

## Results

The online survey was distributed to 597 members of the UK Trial Manager Network (UKTMN) and 159 NIHR Chief Investigators. Responses were received from 126 UKTMN members (21%) and 74 (47%) Chief Investigators. Results will be presented and discussed, including key performance factors and the themes identified.

## Discussion

This survey presents an overview of current practice for choosing sites in UK non-commercial trials. Results will contribute to developing a ‘tool’ to improve site selection.

